# Serum Microelements in Early Pregnancy and their Risk of Large-for-Gestational Age Birth Weight

**DOI:** 10.3390/nu12030866

**Published:** 2020-03-24

**Authors:** Małgorzata Lewandowska, Jan Lubiński

**Affiliations:** 1 Medical Faculty, Lazarski University, 02-662 Warsaw, Poland; 2 Division of Gynecological Surgery, University Hospital, Polna 33 Str., 60-535 Poznan, Poland; 3 Department of Genetics and Pathology, International Hereditary Cancer Center, Pomeranian Medical University, 71-252 Szczecin, Poland; lubinski@pum.edu.pl

**Keywords:** selenium, birth weight, antioxidants, microelements, pregnancy, obesity, fetus, fetal sex

## Abstract

Excessive birth weight has serious perinatal consequences, and it “programs” long-term health. Mother’s nutritional status can be an important element in fetal “programming”; microelements such as selenium (Se), zinc (Zn), copper (Cu), and iron (Fe) are involved in many metabolic processes. However, there are no studies assessing the relationship of the microelements in the peri-conceptual period with the risk of excessive birth weight. We performed a nested case control study of serum microelements’ levels in the 10–14th week of pregnancy and assessed the risk of large-for-gestational age (LGA) newborns using the data from a prospective cohort of pregnant women recruited in 2015–2016 in Poznań, Poland. Mothers delivering LGA newborns (n = 66) were examined with matched mothers delivering appropriate-for-gestational age (AGA) newborns (n = 264). Microelements’ levels were quantified using mass spectrometry. The odds ratios of LGA (and 95% confidence intervals) were calculated by multivariate logistic regression. In the whole group, women with the lowest quartile of Se had a 3 times higher LGA risk compared with women in the highest Se quartile (AOR = 3.00; *p* = 0.013). Importantly, the result was sustained in the subgroup of women with the normal pre-pregnancy BMI (AOR = 4.79; *p* = 0.033) and in women with a male fetus (AOR = 6.28; *p* = 0.004), but it was not sustained in women with a female fetus. There were no statistical associations between Zn, Cu, and Fe levels and LGA. Our study provides some preliminary evidence for the relationships between lower serum Se levels in early pregnancy and a higher risk of large-for-gestational age birth weight. Appropriate Se intake in the periconceptual period may be important for optimal fetal growth.

## 1. Introduction

Studies conducted in recent decades have shown that the intrauterine environment can affect long-term health, and the importance of the role of mother’s nutrition for fetal development and for health “programming” is increasing [1,2,3]. Fetal obesity is one of the most important pregnancy complications. Excessive fetal growth increases the risk of maternal and neonatal perinatal trauma [4,5], and it may “program” (in the child) some adverse health effects in the future (e.g., obesity, diabetes, and even cancer) [1,6,7]. Excessive birth weight includes two categories: large-for-gestational age (LGA) birth weight, defined as a weight over the 90th percentile for a given gestational age and fetal sex; and fetal macrosomia, when the predicted fetal weight exceeds 4000 g (or 4500 g, depending on sources), regardless of gestational age [4,5]. Mechanisms of fetal obesity are not fully understood. However, the mother’s nutritional status and fetal placental circulation as well as genetic factors play a fundamental role in fetal development [8,9]. 

In animal fetal studies and general population studies in humans, obesity has been linked to, among other factors, oxidative stress and inflammation, insulin resistance, and hormonal disorders [3,9,10]. It has been shown that maternal chronic inflammation can increase glucose and lipid availability in the fetus, which results in its excessive growth and fat deposition [11]. Relationships between higher levels of maternal inflammation markers and higher birth weight and obesity in the offspring have been found [12,13]. Some nutrients, including trace elements, have a strong impact on these metabolic disorders, and therefore they may contribute to fetal “programming”. Microelements such as selenium (Se), zinc (Zn), copper (Cu), and iron (Fe) have anti-inflammatory and antioxidant properties, and their deficiency or excess can increase inflammation and oxidative stress [14,15]. These microelements are involved in insulin signal cascade regulation, glucose metabolism, and the functions of many hormones [11,16,17,18]. They also affect cell proliferation and differentiation [2,14,19]. Yet, the role of individual micronutrients in fetal development remains unclear and poorly investigated, and the number of studies on humans is limited [3].

Prospective studies assessing the relationship of Se, Cu, Zn, and Fe in early pregnancy with the risk of high or low birth weight are few and concern the risk of low birth weight [20,21,22]. The results of studies on retrospective associations between the maternal status of these microelements and birth weight are divergent [23], but the levels of microelements in women in late pregnancy or after childbirth may be the attributed to various already developed disorders such as the development of gestational diabetes or pregnancy-induced hypertension; an important element of these disorders is increased oxidative stress, which may result in abnormal levels of trace elements involved in oxidative balance [14,15]. In our earlier case-control study based on data from a prospectively collected cohort of pregnant women without chronic diseases, we found links between lower serum Se levels at the 10–14th weeks of pregnancy and a higher risk of small-for-gestational-age birth weight (SGA) [20]. However, other studies have shown that the relationship between lower levels of trace elements and SGA are inconsistent [24], and meta-analyses have shown that the relationship between multivitamin supplementation in pregnancy and the reduction of SGA risk are weak [25]. Choi et al. found no relationship between lower microelement levels (taking into consideration all the trimesters of pregnancy) and higher SGA risk; on the contrary, lower levels of Se were associated with higher birth weight [24]. 

In the general population (in children, adolescents, and adults), lower levels of Se, Zn, and Fe and higher levels of Cu have been found to be associated with obesity [26,27,28,29]. Experimental studies have found a relationship between a deficiency of selenium in the diet of pregnant animals and low fasting glucose with simultaneous glucose intolerance in the offspring, including the sexual dimorphism of the results found [1]; this may indicate the complexity of the relationship between micronutrients and the weight of newborns (and the risk of LGA or SGA). It is worth noting that in studies of pregnant women, relationships between lower serum levels of Se [30,31], Fe [32], and Cu [33] in early pregnancy and higher risk of such complications as pregnancy-induced hypertension were found. Importantly, low levels of selenium are found in the soil and diet in many regions of the world, resulting in low levels of selenium in humans [34].

A small number of studies and inconsistent results suggest the need to study the relationship between the status of microelements in early pregnancy and the risk of fetal obesity.

To our knowledge, this is the first such prospective study on trace elements as risk markers of excessive birth weight focusing on human subjects. The aim of this study was to assess the relationships of four microelements (Se, Zn, Cu, and Fe) at the end of the first trimester with the risk of LGA. Since maternal obesity is an important LGA risk factor, the subgroups of women with the normal pre-pregnancy body mass index (BMI, with and without gestational diabetes) were examined separately. Women with a male and female fetus were also assessed separately. 

## 2. Materials and Methods 

### 2.1. Study Population and Method

The original cohort wherein the study was nested consisted of 662 women, and 66 (10%) LGA newborns and 548 AGA newborns were found (a total of 750 women were recruited, and 5 cases of miscarriage, 1 case of delivery before the 25th week, and 82 women due to incomplete data were excluded).

The cohort was recruited in 2015–2016, pregnancy results were collected in 2016–2017, and the analysis of data was conducted in 2017–2019. The study was performed at the University Hospital in Poznan, Poland (obstetric–gynecological and neonatological center, with 6000–8000 births a year) and was conducted according to the Helsinki Declaration. It was approved by the Bioethics Committee of the Medical University of Poznan, Poland (approval number 769/15). All the participants signed the Informed Consent Form. 

The inclusion criteria for the cohort were as follows: white women of Polish descent from one region (Wielkopolska), aged 18–45 years at conception, in the 10th (+0) to 14th (+6 days) week of a single pregnancy without aneuploidy and with delivery of a phenotypically normal child ≥ 25 weeks, healthy at recruitment. The exclusion criteria were chronic diseases (with the exception of belonging to the excessive BMI range group), especially pre-pregnancy diabetes mellitus and hypertension, thromboembolism, immunological, and inflammatory diseases, as well as kidney or liver diseases.

The participants of this study were recruited from pregnant women taking typical laboratory tests in the first half of their pregnancy. The data was collected from a questionnaire during recruitment in the 10–14th week of pregnancy (the participants filled it out themselves, but in the presence of midwives). We collected the information such as age, history of pregnancy and parity, gynecological histories, values of blood pressure before recruitment, concurrent diseases, socioeconomic and demographic characteristics, supplements and medications, family medical histories, smoking, and alcohol consumption (all the women declared consuming no alcohol in pregnancy). Mothers’ blood samples were taken at recruitment (in the 10–14th week of pregnancy) from the participants, who reported fasting. Sera were stored at −80 °C. 

After the end of pregnancy, the data on the outcomes and complications were taken from the medical records. The report about pregnancy outcomes included, among others, information such as the infant’s gender, gestational age, birth weight, Apgar scores performed at 1 and 5 min after birth, as well as pregnancy complications such as pregnancy-induced hypertension or gestational diabetes mellitus.

The primary outcome of this analysis was the delivery of a child with large-for-gestational age birth weight (LGA) (compared to appropriate-for-gestational age birth weight (AGA)). The aim of this study was to assess the risk of LGA for levels of maternal microelements measured in serum from the 10–14th gestational week (sera were stored at −80 °C).

The minimal sample size for the original cohort was calculated using the formula for a single proportion:

N = Z^2^/d^2^ × p(1 − p) (1)

(for confidence intervals = 95% and Z = 1.962 = critical value of the normal distribution at α/2, α = 5%, d = margin error, p = disease proportion). In the literature, the frequency of excessive fetal growth was estimated at 3%–15% [5]. For the proportion of p = 15% and margin error d = 3%, the estimated minimal sample size was 544. For the LGA proportion of p = 10% (in this study) and margin error d = 3%, the estimated minimal sample size was 384. The sample collected was large enough to reflect the population with the smallest possible error, as we have shown by calculating the minimum sample size. We did not do a pilot study; therefore, we did not determine the required sample size based on the test power.

The current study was nested in the original cohort. In the current analysis, the study group covered mothers giving birth to LGA infants (n = 66), and the control group covered matched mothers giving birth to AGA infants (n = 264). Four AGA controls were selected for each woman with an LGA newborn (4:1) in relation to the following criteria: mother’s age (±2 years), pre-pregnancy BMI (±10%), and gestational age at delivery (±1 week).

### 2.2. Lifestyle-Related and Socio-Demographic Variables

Pre-pregnancy weight was self-reported. Maternal pre-pregnancy BMI (body mass index) was calculated for each woman as ratio of the weight (kg) and a height^2^ (m^2^) and BMI in the normal range was defined as 18.5–24.99 kg/m^2^. Overweight was defined as 25.0–29.99 kg/m^2^, and obesity was defined as ≥30 kg/m^2^. 

Gestational weight gain (GWG) was defined as the difference between the weight before delivery (data from the Medical Records) and declared pre-pregnancy weight.

Among socio-economic indicators, maternal education included elementary, vocational, secondary, and higher education (education <12 years included elementary and secondary education). 

### 2.3. Pregnancy Outcomes and Complications

The gestational age was estimated based on the fetal first trimester ultrasound measurements. Crown–rump length (CRL) measurements were used for pregnancy dating in 10 (+0) and 13 (+6 days) gestational weeks. Beyond 14 (+0) weeks’ gestation, gestational age estimates are based on the measurement of the head circumference (HC), biparietal diameter (BPD), abdominal circumference (AC), and femur length (FL) or a combination of measurements. 

The birth weight was measured after birth using an electronic scale (in grams). For the assessment of birth weight categories (in percentiles), data from fetal biometry (between 20 and 42 weeks of gestation) for the Polish population for gestational age and the gender of the newborn was used [20]. Large-for-gestational age birth weight (LGA) was defined as a weight >90th percentile. Appropriate-for-gestational age birth weight (AGA) was defined as a weight between the 10th and 90th percentile. Small-for-gestational age birth weight (SGA) was defined as a weight < 10th percentile.

Gestational diabetes mellitus (GDM) was diagnosed (in accordance with the International Association of Diabetes and Pregnancy Study Groups) based on a 2-h 75 g oral glucose tolerance test (OGTT) after an overnight fast, which was conducted between the 24th and 28th week of gestation. GDM-1 was diagnosed when modification of the diet was sufficient to control glucose levels, and GDM-2 was diagnosed when additional insulin therapy was required.

Pregnancy-induced hypertension (PIH) was defined as arterial pressure equal to and higher than 140/90 mmHg (on two occasions, at least 4 h apart, with an oscillometric device and in a sitting position) developed de novo after the 20^th^ week of pregnancy, receding up to 12 weeks after delivery and including two forms: gestational hypertension and preeclampsia.

### 2.4. Risk Factors/Confounding Variables

Risk factors for birth weight/LGA [35] and related to the concentrations of microelements [28,34] have been identified based on the literature. Factors included in this study are maternal age, prior fetal macrosomia, maternal height (height > 170 cm), pre-pregnancy body mass index (BMI), gestational weight gain, parity, smoking, folic acid, and other multivitamin supplementation (preparations containing vitamins and microelements), education < 12 years, gestational age at childbirth, fetal sex, diabetes mellitus, and pregnancy-induced hypertension at present pregnancy, as well as gestational age at recruitment. 

To eliminate the impact of several factors on the estimated LGA risk, the LGA and control group were matched in relation to maternal age (±2 years), pre-pregnancy BMI (±10%), and gestational age at delivery (±1 week) (Section 2.1). Finally, risk factors that differ statistically insignificantly between groups were as follows: mother’s age, pre-pregnancy BMI, and gestational age at delivery (matched variables) as well as parity, smoking, education < 12 years, and folic acid and multivitamin–microelement supplementation for pregnant women.

The risk factors that differ statistically significantly between groups were used as confounding variables: prior fetal macrosomia, maternal height > 170 cm, and gestational weight gain per week.

### 2.5. Determinations of Microelements

Microelements’ concentrations were measured in maternal serum after the end of pregnancy and after selecting the study group and control group. Mothers’ blood samples were taken at recruitment (in the 10–14th week of pregnancy) from women who reported fasting. Venous blood was collected into a Sarstedt (Sarstedt, Nümbrecht, Germany) Monovette system using Serum Z/7.5 mL tubes.

The tubes were incubated at room temperature for at least 30 min (no longer than 2 h) to clot; next, blood samples were centrifuged at 1300 G for 12 min. After that, sera were stored at −80 °C until analysis. Before performing analysis, maternal sera were thawed, vortexed, and centrifuged (at 5000 G for 5 min) before microelement determination. Cases (LGA) and control (AGA) subjects were tested alternately.

The concentrations of microelements in the maternal serum were determined by the inductively coupled plasma mass spectrometer: NexION 350D (PerkinElmer, Shelton, CT, USA). Before an analytical run, the spectrometer was tuned to achieve the manufacturers’ criteria. Methane was used as a reaction gas.

The instrument was calibrated using an external calibration technique. Calibration standards were prepared from 10 µg/mL of Multi-Element Calibration Standard 3 (PerkinElmer, Shelton, CT, USA) by diluting with blank reagent to the final concentrations of 1, 5, and 10 µg/L (for Zn and Cu measurement); 0.1, 0.5, 1.0, 2.0, 5.0, and 10 µg/L (for Se measurement); and 1, 5, 10, and 50 µg/L (for Fe measurement). Correlation coefficients for calibration curves were always greater than 0.999.

Analysis protocol assumed 100-fold dilution of serum in the blank reagent. The blank reagent consisted of 10 mL of 65% Suprapur Grade nitric acid (Merck, Darmstadt, Germany) and 0.20 mL of Triton X-100 (PerkinElmer, Shelton, CT, USA) filled to the mark of a 1-L flask with > 18 MΩ/cm2 deionized water (Merck Millipore, Burlington, MA, USA). The germanium isotope (Ge74) was set as internal standard.

The accuracy and precision of measurements were tested using certified reference material (CRM), Clincheck Plasmonorm Serum Trace Elements Level 1 (Recipe, Munich, Germany). Additionally, internal quality control samples were measured during analysis. General precision was lower than 5% RSD (relative standard deviation). The final concentration included a dilution factor and coefficient that was the mean value of two flanking certified reference materials concentrations divided by the mean concentration determined by the manufacturer of CRM [20,30,32,33]. 

Additional information. The dilution was obtained during previous experiments of our laboratory. Such a high dilution factor (100 times) provides better accuracy and precision due to the elimination of non-spectral interferences i.e., Matrix effects. The limit of detection (LOD) for Se was 0.10 µg/L; for Fe, it was 0.99; for Zn, it was 0.81 µg/L; and for Cu, it was 0.92 µg/L. The LOD was calculated as 3 × SD of 20 measurements of blank. All the results obtained for reference materials were within the manufacturers’ range. The sample-to-sample precision was < 5% RSD (I mean the results of the reference material). Internal Quality Control samples were also within the given range. The final concentration was obtained by multiplication of the results by 100. CRMs were measured every 10 samples. The mean of two flanking results of CRM was divided by the mean value provided from the manufacturer of CRM, which is given as a coefficient. This coefficient was multiplied by the result. Below, we described the method of calculations (Result × 100 × Coefficient = Final Result): CRM = 7.0; Sample 1: 4.8; Sampler 2: 5.6; …; CRM = 7.2; Mean of CRM = 7.1; Manufacturer mean of CRM = 6.6; Coefficient = 6.6/7.1 = 0.929; Final result for Sample 1: 4.8 × 100 × 0.929 = 445.92.

### 2.6. Statistical Analyses

The Shapiro–Wilk test was used to assess the normality of data distribution (the distributions were not normal). The Mann–Whitney U test was used for comparisons of continuous variables between groups (microelements’ concentrations and maternal characteristics). The Pearson’s chi-square test was used for categorical variables’ comparisons. In both tests, the *p*-value < 0.05 was considered to be significant. Data were analyzed by Statistica 13 package. 

In this analysis, the study (LGA) group consisted of 66 women, and the control (AGA) group consisted of 264 matched women. Four AGA controls were selected for each woman with an LGA newborn (4:1) in relation to the following criteria: mother’s age (±2 years), pre-pregnancy BMI (±10%), and gestational age at delivery (±1 week). The whole group (N = 330) and the subgroups of women with the normal pre-pregnancy BMI (with and without gestational diabetes) were examined separately, because maternal obesity or being overweight is an important LGA risk factor. Additionally, women with a male and female fetus were examined separately.

Since the matching of the groups (the LGA and AGA groups) in terms of maternal age, pre-pregnancy BMI, and gestational age of delivery was not ideal (the *p*-value was not equal to or close to 1.0), the associations between different levels of each microelement (Se, Zn, Cu, or Fe, separately) and LGA risk were evaluated by logistic regression, in which variables that did not differ statistically significantly were not used as disturbance variables (Section 2.4). The crude odds ratios (OR) of LGA (and 95% confidence intervals CI) for microelement levels were calculated in univariate logistic regression. The adjusted odds ratios (AOR) of LGA (and 95% confidence intervals, or CI) were calculated in multivariate logistic regression after adjusting for confounders (risk factors that were statistically different between the groups) as described above in Section 2.4.

For the assessment of associations of each microelement with LGA occurrence, the data set (in the whole group or subgroups) was divided into equal quartiles (Q1, Q2, Q3 and Q4) based on the distribution of the concentrations of each maternal microelement (from the lowest to the highest concentrations). The number of mothers with LGA infants (and AGA infants) was calculated in each quartile. The odds ratios (OR and AOR) were calculated for each quartile with regard to the reference quartile (the quartile with the lowest number of LGA cases; OR = 1.00). The *p*-value for odds ratios was assessed using the Wald test, and *p* < 0.05 was considered to be significant.

Box and whisker plots for maternal selenium (Se) concentrations and a graph of the risk profile of LGA for all values of Se concentrations were presented.

## 3. Results

The general characteristics of participants in the study (LGA) group (n = 66) and matched control (AGA) group (n = 264) are presented in Table 1. The women were healthy at the recruitment (in the 10–14th week). The mean age of the women in the LGA group was 36.0 years (range 26–43), and in the AGA group, it was 35.7 years (range 25–44) (*p* = 0.556). The differences between the groups were not statistically significant in terms of several risk factors of LGA and microelements’ levels. The pre-pregnancy BMI (a recognized risk factor of excessive fetal growth) was not statistically different between the LGA versus AGA group (*p* = 0.291), and the differences were not statistically significant in terms of the number of women with gestational diabetes mellitus (*p* = 0.117).

Several risk factors were statistically different between the groups. The number of women with height > 170 cm (*p* = 0.046), gestational weight gain/week (*p* < 0.0001), and number of women with prior fetal macrosomia (*p* = 0.002) were higher in the LGA group compared to the controls. 

The comparison of maternal microelements between the LGA and AGA group in the whole group and subgroups are presented in Table 2. The mean serum concentrations (and standard deviation) are included. The mean values of all the elements were lower in women in the LGA group compared to the control (AGA) group; however, the difference was statistically significant for selenium (Se) only. This result was found in the whole group and the subgroup of women with the normal pre-pregnancy BMI (with and without gestational diabetes) as well as in the women with a male fetus (Table 2).

**Figure 1:**
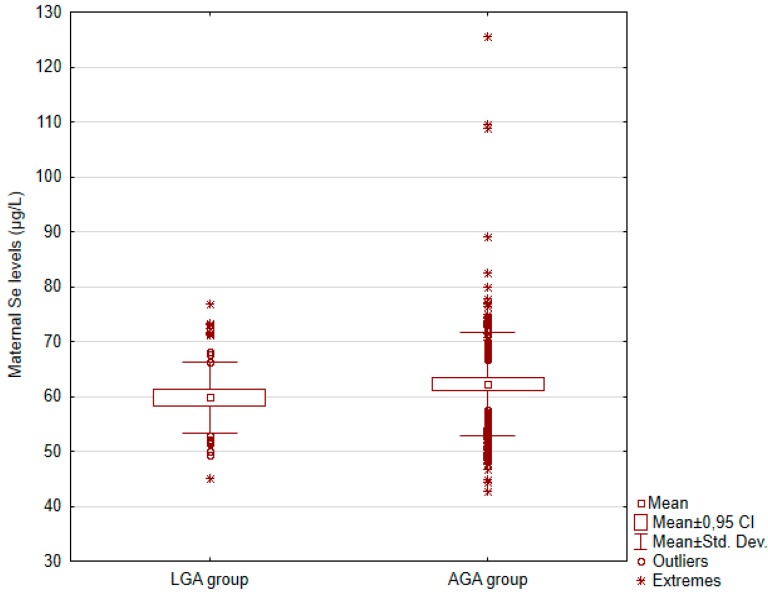
Box and whisker plots for maternal selenium concentrations in the LGA and AGA group, in the whole group; CI: confidence intervals (95%); Std. Dev.: standard deviation.

Figure 1 shows box and whisker plots for Se concentrations in the LGA and AGA group, in the whole group.

There were no statistical associations between microelements and LGA in other subgroups (women with BMI ≥ 25 kg/m^2^ and women with a female fetus) (Table S1).

Lower levels of Se were found in the subgroup of women with the pre-pregnancy BMI ≥ 25 kg/m^2^ compared to normal BMI (*p* < 0.001), as well as in the women with a female fetus compared to the women with a male fetus (*p* = 0.121), and the LGA proportion was similar between all subgroups (Table S2).

The risk of LGA and the number of LGA cases in each quartile of concentrations of the microelements are presented in Table 3 and Table S3. A significant difference in the number of LGA cases between quartiles was found only in the selenium (Se) analyses (Table 3). The highest Q4 quartile of Se concentrations was the reference quartile (with the lowest number of LGA). In the whole group, the women in the lowest (Q1) quartile of Se (≤55.88 µg/L) had a 3-fold increase in the LGA risk compared to women in the highest (Q4) quartile (>66.01 µg/L), and the adjusted odds ratio was AOR = 3.00 (*p* = 0.013) after adjusting for maternal height > 170 cm, gestational weight gain per week, and prior fetal macrosomia. The results were sustained in the subgroup of women with the normal pre-pregnancy BMI (AOR = 4.79; *p* = 0.033) and the women with a male fetus (AOR = 6.28; *p* = 0.004), but not in the subgroup of BMI ≥ 25 kg/m^2^ and the women with a female fetus (Table S3). Table S3 also shows the results for all microelements in the whole group. 

**Figure 2:**
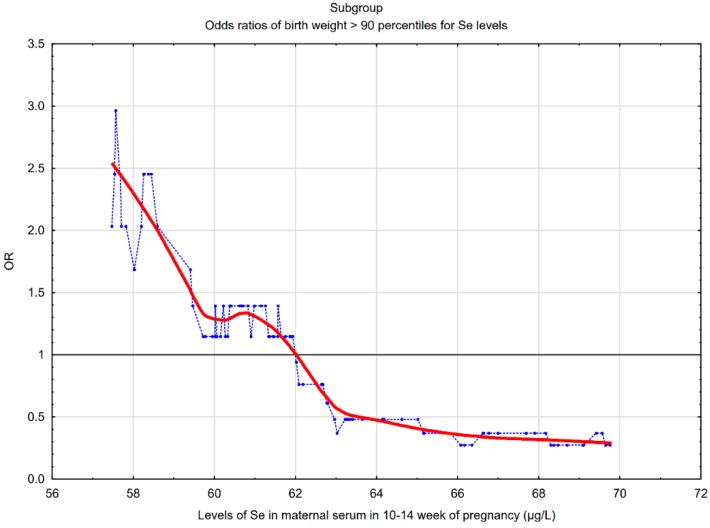
The risk of LGA birth weight (>90th percentile) for early pregnancy maternal serum selenium (Se) concentrations in the subgroup of women with the normal pre-pregnancy BMI (N = 145). The graph shows the changes in the odds ratio of LGA calculated on a sliding window with respect to the changes in the Se concentrations. The window width adopted was 30 observations. The blue points correspond to the odds ratios (OR) of LGA in a window containing a fixed number of neighboring cases (the center of the window is for a given value of Se concentration). The red curve represents the LGA risk profile smoothed with the Lowess method. The horizontal black line is the reference line for OR = 1; the points above the line indicate an increased risk.

The picture in Figure 2 shows that the lower maternal serum Se concentrations at the end of the first trimester were associated with a higher risk of LGA in the subgroup of women with the normal pre-pregnancy BMI. In the subgroup, the threshold point was 62.0 µg/L, below which an increase in the odds ratios of LGA was observed. In the whole group, the threshold point was 63.0 µg/L.

In the supplement, the risk profile of SGA (small-for-gestational age birth weight) in the subgroup of women with the normal pre-pregnancy BMI was also presented (Figure S1). The results were obtained in our previous case control study based on the results of the same cohort [20]. This chart has not been previously presented. 

Table S4 shows a short amount of characteristics of the participants of two studies: the current case control study for LGA risk and the previous case control study for SGA risk [20]. Differences in the structure of the studied groups in terms of pre-pregnancy BMI and smoking were found (Table S4). 

## 4. Discussion

The main result of our study was finding relationships between lower maternal Se levels at the end of the first trimester and a higher risk of LGA.

Horan et al. found that maternal selenium intake in the first trimester was negatively associated with the subscapular/triceps skinfold ratio (the ratio for estimating neonatal adiposity). Trimester 3 selenium intake was negatively associated with abdominal circumference [11]. Additionally, studies in animals have shown that maternal antioxidant supplementation reduced the effect of mother’s obesity on fetal adiposity [36]. Lower Se levels have been shown to be associated with obesity in children, adolescents, and adults [26,28]. In our study, a statistically lower level of Se was also found in women with pre-pregnancy BMI ≥ 25 kg/m^2^ compared to women with the normal BMI (*p* = 0.0001) (Table S1). On the other hand, experimental studies by Hofstee et al. have shown a relationship between a deficiency of selenium in the diet in pregnant mice and low fasting glucose with simultaneous glucose intolerance in the offspring (including sexual dimorphism of the results) [1]. In a prospective study of mothers in the second trimester and their 4–6-year-old children, Kupsco et al. found relationships between higher selenium levels in maternal whole blood and lower plasma triglycerides in children [37].

The literature data indicate a link between lower Se levels in the first half of pregnancy and SGA [20,21,22], and it is explained by a deficiency of selenium antioxidant properties during placental development, leading to placental dysfunction [15]. Importantly, in our previous case control study based on data from the same prospectively collected cohort of pregnant women, we found links between lower serum Se levels at the 10–14th week and a higher risk of SGA [20]. In our previous study (for SGA risk), the threshold point was 56 µg/L, below which an increase in the risk of SGA was observed [20], and in a subgroup of the normal pre-pregnancy BMI, the threshold point was 58.5 µg/L (Figure S1). In the current study, the threshold point was 62.0 µg/L, below which an increase in the odds ratios of LGA was observed (Figure 2). In our two studies (for LGA and SGA risk), we obtained different threshold points, but not the difference of a few µg/L that is important here. We want to emphasize that the LGA and SGA risk profiles were different. In the LGA study, the two lower quartiles (Q1 and Q2) were associated with a higher LGA risk (Table 3 and Table S3). In the SGA study, only the lowest concentrations (in the lowest Q1 quartile) were associated with a higher SGA risk. Bogden et al. also found the relationship between the lowest level (the lowest tertile) of Se concentrations and the higher SGA risk [22]. However, other studies have shown that the relationship between lower levels of trace elements and SGA are inconsistent [24]. Choi et al. found no relationship between lower Se levels (taking into consideration all the trimesters of pregnancy) and higher SGA risk; on the contrary, lower levels of Se were associated with higher birth weight [24,25]. 

In our two studies, the groups (for LGA and SGA risk) differed in terms of BMI and smoking (Table S4). In accordance with the literature, obesity and smoking are factors that strongly lower Se levels [26,28,30]. However, both of these factors should not affect our main score in both our studies (for LGA and SGA risk). Firstly, the number of smokers was small and did not differ statistically between cases and their controls (in both studies) (Table 1) [20]. Secondly, pre-pregnancy BMI did not differ statistically significantly between cases and their controls (in both studies) (Table 1) [20]. Most importantly, our main result was statistically significant in subgroups of women with pre-pregnancy BMI within the normal range (in both studies). 

The development of these two outcomes (LGA and SGA) may depend on the coexistence of other factors, and this requires further investigation. Low levels of Se and increased oxidative stress can be a factor associated with abnormal placental development and a higher risk of SGA [15]. However, lower levels of Se in combination with other obesogenic factors may increase the risk of LGA [9]. 

In the current study, the recognized factors increasing the risk of LGA [35] and potential factors affecting microelements’ levels [28,34] were matched or statistically insignificantly differ between the LGA and control (AGA) group or were used to correct the risk as confounders. Matching the mothers’ characteristics in our study has reduced the impact of several risk factors on results (Table 1). Pre-pregnancy obesity (BMI) and gestational diabetes (GDM) are listed among the most important risk factors for neonatal LGA [9,33]. Gestational diabetes mellitus (GDM) did not increase the LGA risk in our group; however, our groups were matched to pre-pregnancy BMI and age (which are GDM and LGA risk factors). Our studied groups were also matched to gestational age at delivery. We recruited women without chronic diseases, so pre-existing diabetes and diseases with inflammatory states were excluded.

The differences in Se concentrations between the LGA and AGA (control) groups were statistically significant but not large across the whole group (*p*-value was 0.036). However, after adjusting for all the confounders, the adjusted odds ratio of LGA for the lowest Se quartile compared to the highest quartile was AOR = 3.00 (*p* = 0.013), and our results deserve further investigation. The outlined odds ratio profile (Figure 2) indicates a clear increase in LGA risk while reducing Se concentrations. Importantly, the incidence of LGA in various subgroups was similar (*p* > 0.05) (Table S2).

Our results were sustained (and were stronger) in the subgroups of women with the normal pre-pregnancy BMI (with and without gestational diabetes, or GDM). Women with the lowest quartile of Se (Q1 quartile) compared with women in the highest (Q4) quartile had a 4.79 times higher LGA risk in the subgroups of women with the normal pre-pregnancy BMI (AOR = 4.79; *p* = 0.033), and a 1.46 times higher LGA risk (AOR = 1.46; *p* = 0.506) in the subgroups of women with the pre-pregnancy BMI ≥ 25 kg/m^2^ (Table S3). We believe that the BMI difference between the subgroups is the main reason for the negligible result in the subgroup ≥ 25 kg/m^2^. A lower Se concentration among AGA controls in this subgroup (Table S2) was found (which is consistent with the literature data and our result), resulting in a smaller concentration difference between cases and controls (Table S2), which is the reason for the lower OR/AOR [26,28,30]. This suggests that the difference in study populations in terms of BMI may be the reason for discrepancies between studies.

Our results were also sustained in women with a male fetus (AOR = 6.28; *p* = 0.004 for Se in Q1 quartile versus Q4). The result in women with a female fetus was insignificant (AOR = 1.20; *p* = 0.791) (Table S3). In the subgroup of women with a female fetus, a lower Se concentration among AGA controls was found, resulting in a smaller concentration difference between cases and controls (Table S2), and this is the cause of a lower OR/AOR. In this subgroup, higher frequencies of BMI ≥ 25 kg/m^2^ and smokers were found, which could have resulted in lower Se concentrations. These two factors (obesity and smoking) lower Se levels (according to the literature data) [26,28,30]. 

The limitations in our study can be the tubes used to collect blood for serum. They are not recommended for trace elements, although they are used in many tests, and we have screened lots of the tubes used in this examination.

The mechanisms of fetal obesity development remain unexplained, but there are factors that can link lower levels or deficiency of selenium (Se) with this pregnancy outcome also in women without obesity and with well-controlled glucose levels. Firstly, the intrauterine (fetal) phase of human development is influenced by many factors, and the placenta forms a special interface between the mother and the fetus [3,8,9]. The placenta is structurally and functionally adaptable, which helps alleviate some of the risks, e.g., nutrients deficiency or hypoxia [3]. The heterogeneity of the placental response to maternal obesity in relation to the birth weight of the newborn was also found, indicating a possible contribution of various additional factors (e.g., metabolic factors or those regulating the phenotype of placental transport) [3]. Secondly, obesogenic factors in the uterus include oxidative stress and inflammation, insulin resistance as well as hyperinsulinemia and hyperglycemia, and other factors such as triglycerides and free fatty acids [3,9,38]. It has been shown that maternal chronic inflammation can increase glucose and lipid availability in the fetus, which results in its excessive growth and fat deposition [11]. Previous studies have shown that the pro-inflammatory state preferentially changed the fetal mesenchymal stem cell differentiation from a myogenic state to an adipogenic state [9,39]. Relationships between higher levels of maternal inflammation markers (e.g., CRP or IL-6 and TNF-alpha inflammatory cytokines) and higher birth weight and obesity in the offspring have also been observed [12,13]. Studies of the general population in animals and humans also showed that processes related to the development of obesity are multifactorial and complex, and inflammation and oxidative stress have been associated with obesity [40,41].

Selenium involvement in cellular, metabolic, and hormonal functions may be part of the processes listed above. In optimal concentrations, selenium (Se) has strong antioxidant and anti-inflammatory properties [11,14,15]. It works in the body through selenoproteins, which are found in various tissues and show many activities. We know of the antioxidative role of seleno-dependent glutathione peroxidase (GP-x) as well as thioredoxin reductase (Th-Red-x) and the anti-inflammatory action of Selenoprotein P. These enzymes are found in the placenta and together with Cu, Zn-DOS (superoxide dismutase), they form an antioxidant system that protects the tissues of the placenta and the fetus from excessive oxidative stress; with the trophoblast invasion of the walls of the spiral arteries, the blood flow in the placental circulation increases, and so does the oxygen pressure and the concentration of reactive oxygen species (ROS) [15].

Selenium affects the regulation of the insulin signal cascade and glucose metabolism. Selenium is also involved in immunological functions, cell proliferation and differentiation, transcription regulation, and interaction with signal proteins [11,14,19]. However, the associations between selenium levels and glucose metabolism and insulin resistance in humans are divergent; both lower and higher Se levels were associated with type 2 diabetes [1,34]. Recent evidence indicates that oxidative stress (reactive oxygen species (ROS) and stress of the endoplasmic reticulum) and inflammation in the hypothalamus affect its food intake-regulating functions and energy expenditure, and the major antioxidant enzymes in the brain include selenium-dependent GPx and ThRedx, as well as Cu, Zn-superoxide dismutase [40,41,42,43]. Selenium can also affect metabolic disorders through its association with thyroid hormone function [10,44]. Hypothyroxinaemia has been found to be associated with obesity [45], and a meta-analysis by Talebi et al. found significantly lower levels of Se in patients with hypothyroidism compared to healthy people [46].

We want to point out that there is evidence of sexual dimorphism (relative to gene expression) that can modulate responses to environmental factors and affect differences in fetal “programming” [1,3]. Placentas of male fetuses show an inflammatory profile, which may suggest investing in fetal development and less placental reserve capacity in an adverse environment [3]. Female fetus placentas display a higher expression of genes associated with hormonal functions, immune regulation, and placental growth (indicating investing in placental structure and function) [3]. Gender differences in gene expression may cause different placental responses to low or high-fat diets, hypoxia, or glucocorticoids. 

Our result shows the relationship between a lower level of Se and a higher risk of LGA, but it does not explain the mechanism of LGA development; this mechanism is complicated and multifactorial. Our result can only suggest that the lower status of Se and the deficiency of its actions promote obesogenic factors. Confirming our result would allow the implementation of targeted dietary interventions. The level of selenium in the body is influenced by genetic and environmental factors and the availability of this element in the diet. Low levels of selenium in soil and in the diet as well as low levels of Se in humans are found in many regions of the world, e.g., in Central and Eastern Europe. Despite various interventions to enrich the diet with selenium, there are geographical differences in its status in humans. Low levels of Se are recorded in Poland; average values are 70 µg/L, but in some regions of the country, they are as low as 50–55 µg/L [47]. Demand requirements indicate that the Se level should be 70–90 µg/L to ensure adequate glutathione peroxidase (GP-x) activity [34]. Optimal selenium levels are estimated at 60–120 µg/L [47], and all studies showing relationships of lower Se levels with adverse health outcomes are valuable. The effects of Se on human health (including glucose metabolism) show a “U” curve because both the deficiency and excess of Se are harmful. Therefore, selenium supplementation should only take place after their deficiency is confirmed. 

In our study, the concentrations of Zn, Cu, and Fe in maternal serum in the 10–14th week were not associated with LGA risk, although these microelements are also associated with metabolic disorders, such as insulin resistance as well as diabetes mellitus and obesity [16,17,18]. In their optimal levels, they also have antioxidant and anti-inflammatory properties, and their deficiency or excess may increase inflammation and oxidative stress [15]. Other prospective studies showed no associations between SGA risk and maternal Zn and Fe or Cu levels [20,21]. Dietary deficiencies in Zn intake are currently not a problem in developed countries, which may explain the lack of association between maternal Zn levels and abnormal fetal weight. An important element in iron studies is the variety of Fe status markers (e.g., serum ferritin, soluble transferrin receptor, and others). Due to the presence of inflammation and oxidative stress in pregnancy, interpreting the results of markers such as ferritin (an acute phase protein) can be difficult.

### Advantages and Limitations

Our study has several strengths. The study was nested in a prospective cohort of pregnant women, and only such a model allows the assessment of risk markers. Study participants were healthy at recruitment (and at serum sampling) and chronic diseases associated with inflammation state and/or oxidative stress were excluded. We performed a detailed analysis by quartiles. A graphic picture of the risk of LGA for many Se concentrations in early pregnancy was presented. We matched the study groups well, which allowed ruling out the influence of several confounders. We have evaluated the entire group and the subgroup of women with normal BMI separately. We showed the results for Se against the background of other microelements involved in the inflammatory processes and oxidative balance. The evidence obtained is important because of the relationship between excessive fetal growth and “programming” long-term health. 

We are aware of the limitations of the study. Patients reported their pre-pregnancy weight on their own. Longitudinal examination would be interesting. We did not study other markers, e.g., markers of glycemic control. There was no information about the degree of glucose control during pregnancy; however, the number of GDM-2 cases (gestational diabetes mellitus treated with insulin) was small (4.2%). The limitations in our study are the tubes used to collect blood for serum; the tubes used in this examination were not certified for trace element content.

## 5. Conclusions and Future Work

This study’s results showed that lower maternal serum selenium (Se) levels in the 10–14th week of the pregnancy were associated with a higher risk of LGA. It is the first study in this topic. The relationships found should be checked in subsequent prospective studies on larger samples. Simultaneous LGA and SGA risk testing is needed.

Confirmation of our result could suggest that women with lower selenium levels could be included in the increased surveillance group. A non-invasive examination of mother’s selenium levels in early pregnancy, and preferably before pregnancy, could be a diagnostic tool, while also showing the direction of dietary changes to reduce the risk of abnormal birth weight. However, well-prepared randomized trials are needed. Appropriate Se intake in the periconceptual period may be important to optimal fetal growth and fetal “programming”.

Further research in this topic could involve assessing offspring results, other ranges of birth weight percentiles, differences between racial and ethnic groups, or different geographical locations.

## Figures and Tables

**Table 1:** The characteristics of mothers in the appropriate-for-gestational age birth weight (AGA) and large-for-gestational age birth weight (LGA) group.

	Controls (AGA Group) * (n = 264)	Cases (LGA Group) * (n = 66)	
Characteristics	Mean (SD) or n (%)	Mean (SD) or n (%)	*p* ***
Maternal age (years)	35.7 (3.8)	36.0 (3.6)	0.556
Maternal age (range)	(25–44)	(26–43)	
Primiparous	87 (33.0%)	20 (30.3%)	0.681
Fetal macrosomia in history	10 (3.8%)	10 (15.2%)	0.002
Maternal height > 170 cm	57 (21.6%)	22 (33.3%)	0.046
Gestational age at recruitment (weeks)	12.2 (0.9)	12.0 (0.8)	0.209
Women who have never smoked	217 (82.2%)	59 (89.4%)	0.220
Education < 12 years (for available data)	26 (11.9%)	6 (10.3%)	0.926
**Vitamins–Minerals**			
Folic acid in I trimester	87 (33.0%)	19 (28.8%)	0.517
Vitamins–Minerals in II–III trimester **	134 (50.8%)	33 (50.0%)	0.912
**Maternal weight**			
Pre-pregnancy BMI (kg/m^2^)	26.2 (4.7)	27.1 (5.4)	0.291
Pre-pregnancy BMI (range)	(18.1–40.4)	(17.8–42.9)	
Normal pre-pregnancy BMI: 18.5–24.99 kg/m^2^	118 (44.7%)	27 (40.9%)	0.579
GWG/week (kg/week)	0.32 (0.16)	0.39 (0.18)	0.001
**Outcomes and Complications**			
Fetal sex/son	139 (52.7%)	37 (56.1%)	0.620
Newborn birth weight (g)	3310.2 (502.3)	4174.1 (254.1)	<0.0001
Gestational age at delivery (weeks)	38.6 (2.1)	38.8 (1.1)	0.813
APGAR-5′ < 7 ****	-	1 (1.5%)	-
Pregnancy-induced hypertension (PIH)	46 (17.4%)	18 (27.3%)	0.070
Preeclampsia (PE)	5 (1.9%)	1 (1.5%)	1.000
Gestational diabetes mellitus (GDM)	56 (21.2%)	20 (30.3%)	0.117
GDM-2	10 (3.8%)	4 (6.1%)	0.491

* AGA: appropriate-for-gestational age birth weight (10–90th percentile), LGA: large-for-gestational age birth weight (>90th percentile); ** multivitamins’ and microelements’ preparations; *** The Mann–Whitney U test was used for comparisons of continuous variables (medians were compared), and the Pearson chi-square test was used for categorical variables comparisons (*p*-value < 0.05 was considered to be significant); **** APGAR scores at 5 min after birth; BMI: body mass index; GWG: gestational weight gain; GDM-1 was diagnosed when modification of the diet was sufficient to control glucose levels, and GDM-2 was diagnosed when additional insulin therapy was required.

**Table 2:** The characteristics of microelements’ concentrations in the AGA and LGA group in the whole group and in subgroups.

	Controls (AGA Group) *	Cases (LGA Group) *	
Microelements (µg/L) **	Mean (SD)	Mean (SD)	*p* ***
Whole group (N = 330)	n = 264	n = 66	
Selenium (Se)	**62.20 (9.37)**	**59.79 (6.48)**	**0.036**
Zinc (Zn)	628.46 (196.27)	616.78 (83.75)	0.880
Copper (Cu)	1786.18 (341.92)	1741.92 (302.37)	0.479
Iron (Fe)	1007.46 (346.43)	976.88 (293.86)	0.735
Women with the normal BMI (N = 145)	n = 118	n = 27	
Selenium (Se)	**63.80 (7.06)**	**60.01 (6.55)**	**0.006**
Zinc (Zn)	613.72 (89.85)	603.43 (80.52)	0.436
Copper (cu)	1676.94 (256.62)	1668.03 (258.96)	0.933
Iron (Fe)	1038.41 (336.96)	1008.84 (317.62)	0.694
Normal BMI without GDM (N = 113)	n = 94	n = 19	
Selenium (Se)	**63.38 (6.65)**	**59.90 (7.05)**	**0.011**
Zinc (Zn)	608.59 (86.63)	611.02 (84.44)	0.911
Copper (Cu)	1658.33 (245.93)	1655.25 (289.50)	0.962
Iron (Fe)	1033.39 (339.41)	1024.61 (324.65)	0.918
Women with a male fetus (N = 176)	n = 139	n = 37	
Selenium (Se)	**63.12 (9.37)**	**59.52 (6.91)**	**0.012**
Zinc (Zn)	623.01 (110.99)	626.42 (88.28)	0.719
Copper (Cu)	1764.86 (359.34)	1776.44 (280.27)	0.594
Iron (Fe)	1035.23 (350.76)	970.93 (333.42)	0.424

* AGA: appropriate-for-gestational age birth weight (10–90th percentile), LGA: large-for-gestational age birth weight (>90th percentile); ** Microelements were measured in maternal serum from the 10–14th gestational week; *** The Mann–Whitney U test was used and medians were compared (*p*-value < 0.05 was considered to be significant); Normal pre-pregnancy BMI: body mass index 18.5–24.99 kg/m^2^; GDM: gestational diabetes mellitus.

**Table 3:** The adjusted odds ratios of LGA (>90th percentile) for maternal selenium levels in the serum at the end of first trimester, in the whole group and subgroups.

			Odds Ratios of LGA (>90th Percentile)	
Quartiles	Se Levels (µg/L)	LGA/AGA	OR * (95% CI); *p* ***	AOR * (95% CI); *p* **
**Whole group** **(N = 330)**				
Q_1_	42.69–55.88	21/61	2.51 (1.10–5.74); **0.029**	3.00 (1.26–7.15); **0.013**
Q_2_	55.88–61.16	20/63	2.32 (1.01–5.32); **0.047**	2.41 (1.02–5.67); **0.044**
Q_3_	61.16–66.01	15/67	1.63 (0.69–3.89); 0.266	1.71 (0.71–4.15); 0.234
Q_4_	66.01–125.54	10/73	1 ***	1 ***
**Women with the normal BMI** **(N = 145) #**				
Q_1_	42.68–58.27	11/25	3.63 (1.03–12.76); **0.044**	4.79 (1.13–20.26); **0.033**
Q_2_	58.27–61.86	8/28	2.36 (0.64–8.66); 0.197	3.00 (0.74–12.28); 0.126
Q_3_	61.86–67.69	4/32	1.03 (0.24–4.48); 0.967	1.46 (0.30–7.09); 0.635
Q_4_	67.69–89.17	4/33	1 ***	1 ***
**Women with a male fetus** **(N = 176)**				
Q_1_	44.39–56.32	16/28	4.42 (1.36–14.34); **0.013**	6.26 (1.79–22.05); **0.004**
Q_2_	56.32–61.46	8/36	1.82 (0.52–6.32); 0.347	1.97 (0.56–7.02); 0.293
Q_3_	61.46–67.32	8/36	1.94 (0.55–6.83); 0.301	2.32 (0.64–8.45); 0.200
Q_4_	67.32–125.54	5/39	1 ***	1 ***

! Selenium concentrations were measured in the serum from the 10–14th week, and border values were included in the lower quartile; * OR: crude odds ratios calculated in the univariate logistic regression in matched groups and AOR: adjusted odds ratio calculated in the multivariate logistic regression after adjusting for maternal height, gestational weight gain per week, and prior fetal macrosomia; ** *p*-value obtained using the Wald test (*p* < 0.05 was considered to be significant); *** the reference quartile with the lowest number of LGA cases (OR/AOR = 1.00); CI: confidence intervals; LGA cases (mothers delivering to newborn > 90th percentile); AGA controls (mothers delivering to newborn between the 10th and 90th percentile). # Subgroup of the normal pre-pregnancy body mass index: 18.50–24.99 kg/m^2^.

## Supplementary Materials

The following are available online at https://www.mdpi.com/2072-6643/12/3/866/s1, Figure S1: The risk profile of SGA for early pregnancy serum Se levels in a subgroup of women with the normal pre-pregnancy BMI, Table S1: The characteristics of microelements’ concentrations in the AGA and LGA group, in the subgroup of pre-pregnancy BMI ≥ 25 kg/m^2^ and the women with a female fetus, Table S2: Characteristics of participants in different subgroups, Table S3: The adjusted odds ratios of LGA birth weight for levels of all microelements in the whole group (N = 330) and for Se levels in the subgroups, Table S4: Characteristics of participants of two studies (that were based for data from the same cohort): for LGA and SGA risk.
