# Supplementary material for: Serum Microelements in Early Pregnancy and their Risk of Large-for-Gestational Age Birth Weight
Source: Nutrients. 2020 Mar 24;12(3):866. doi: 10.3390/nu12030866 (PMC7146262; doi:10.3390/nu12030866)
Supplement: Supplementary file 1 [file nutrients-12-00866-s001.zip › Table S2.docx]

**Table S2.** Characteristics of participants in different subgroups

|  | **Study subgroup** | **Other subgroup** |  |
| --- | --- | --- | --- |
|  | Mean (SD) or n (%) | Mean (SD) or n (%) | p * |
|  | **Normal BMI ****  **(n = 145)** | **BMI ≥25 kg/m²**  **(n = 180)** |  |
| **Selenium concentrations (µg/L) ***** |  |  |  |
| Whole subgroup | 63.10 (7.10) | 60.41 (9.39) | **<0.001** |
| LGA cases | 60.01 (6.55) | 59.94 (6.21) | SI |
| AGA controls | 63.80 (7.06) ** ** | 60.53 (10.07) | **<0.001** |
| **Characteristics :** |  |  |  |
| Number of LGA cases | 27 (18.6%) | 37 (20.6%) | SI |
| Maternal age | 35.9 (3.6) | 35.8 (3.8) | SI |
| Gestational age at recruitment | 12.1 (0.8) | 12.2 (0.9) | SI |
| Gestational age at delivery | 38.8 (1.3) | 38.6 (2.3) | SI |
| Birth weight | 3499.6 (422.4) | 3472.06 (676.4) | SI |
| Primiparous | 41 (28.3%) | 65 (36.1%) | SI |
| FA | 39 (26.9%) | 65 (36.1%) | SI |
| Multivitamins | 74 (51.0%) | 90 (50.0%) | SI |
| Smokers | 15 (10.3%) | 38 (21.1%) | **0.009** |
| GWG above recommendations # | 40 (27.6%) | 106.9 (58.9%) | **<0.001** |
| Education < 12 years | 9 (7.4%) | 23 (15.2%) | SI |
| PIH cases | 15 (10.3%) | 49 (27.2%) | **<0.001** |
| GDM cases | 32 (22.1%) | 43 (23.9%) | SI |
| Fetal sex / son | 85 (58.6%) | 90 (50.0%) | SI |
|  | **Male fetus**  **(n = 176)** | **Female fetus**  **(n = 154)** |  |
| **Selenium concentrations (µg/L) ***** |  |  |  |
| Whole subgroup | 62.37 (9.01) | 60.97 (8.77) | SI |
| LGA cases | 59.52 (6.91) | 60.13 (6.00) | SI |
| AGA controls | 63.12 (9.37) ** ** | 61.17 (9.31) | **0.037** |
| **Characteristics :** |  |  |  |
| Number of LGA cases | 37 (21.0%) | 29 (18.8%) | SI |
| Maternal age | 35.9 (3.8) | 36.6 (3.7) | SI |
| Pre-pregnancy BMI (kg/m²) | 26.1 (4.8) | 26.8 (5.0) | SI |
| Gestational age at recruitment | 12.2 (0.8) | 12.1 (0.9) | SI |
| Gestational age at delivery | 38.6 (2.0) | 38.7 (1.9) | SI |
| Birth weight | 3565.9 (592.0) | 3388.3 (548.9) | **0.003** |
| Primiparous | 55 (31.3%) | 52 (33.8%) | SI |
| FA | 50 (28.4%) | 56 (36.4%) | SI |
| Multivitamins | 89 (50.6%) | 78 (50.7%) | SI |
| Pre-pregnancy BMI ≥25 kg/m² | 90 (51.1%) | 90 (58.4%) | SI |
| Pre-pregnancy BMI : 18.5-24.99 kg/m² | 85 (48.3%) | 60 (39.0%) | SI |
| Smokers | 26 (14.8%) | 28 (18.2%) | SI |
| GWG above recommendations # | 84 (47.7%) | 62 (40.3%) | SI |
| Education < 12 years | 19 (12.8%) | 13 (10.1%) | SI |
| PIH cases | 32 (18.2%) | 32 (20.8%) | SI |
| GDM cases | 45 (25.6%) | 31 (20.1%) | SI |
| * The Mann-Whitney U test was used for comparisons of continuous variables and the Pearson chi-square test was used for categorical variable comparisons (p-value < 0.05 was considered to be significant); **Normal pre-pregnancy BMI: body mass index 18.5 – 24.99 kg/m²; *** Microelements were measured in the serum from the 10-14-th gestational week; ** ** statistically significant differences between the cases and controls were found; # gestational weight gain above the IOM (Institute of Medicine) recommendations; | | | |

SI: statistically insignificant.
