# Supplementary material for: Serum Microelements in Early Pregnancy and their Risk of Large-for-Gestational Age Birth Weight
Source: Nutrients. 2020 Mar 24;12(3):866. doi: 10.3390/nu12030866 (PMC7146262; doi:10.3390/nu12030866)
Supplement: Supplementary file 1 [file nutrients-12-00866-s001.zip › Table S3.docx]

**Table S3.** The adjusted odds ratios of LGA birth weight for levels of all microelements in the whole group (N = 330) and for Se levels in the subgroups.

| **Quartiles** |  | | | **Odds ratios of LGA birth weight (>90^th^ percentile)** | | |
| --- | --- | --- | --- | --- | --- | --- |
| **Whole group**  **(N = 330)** | | **Levels (µg/L)!** | **LGA/AGA** | | **OR* (95% CI); p**** | **AOR* (95% CI); p**** |
| Selenium (Se) | |  |  | |  |  |
| Q_1_ | | 42.69 - 55.88 | 21/ 61 | | 2.51 (1.10-5.74); **0.029** | 3.00 (1.26-7.15); **0.013** |
| Q_2_ | | 55.88 - 61.16 | 20/ 63 | | 2.32 (1.01-5.32); **0.047** | 2.41 (1.02-5.67); **0.044** |
| Q_3_ | | 61.16 - 66.01 | 15/ 67 | | 1.63 (0.69-3.89); 0.266 | 1.71 (0.71-4.15); 0.234 |
| Q_4_ | | 66.01 - 125.54 | 10/73 | | 1*** | 1*** |
| Copper (Cu) | |  |  | |  |  |
| Q_1_ | | 883.61 - 1585.61 | 17/ 65 | | 1.39 (0.63-3.08); 0.420 | 1.30 (0.58-2.950; 0.524 |
| Q_2_ | | 1585.61 - 1746.67 | 20/ 63 | | 1.69 (0.77-3.67); 0.188 | 1.59 (0.72-3.51); 0.253 |
| Q_3_ | | 1746.67 - 1960.76 | 13/ 69 | | 1*** | 1*** |
| Q_4_ | | 1960.76 - 3956.76 | 16/ 67 | | 1.27 (0.57-2.84); 0.564 | 1.36 (0.60-3.10); 0.463 |
| Zinc (Zn) | |  |  | |  |  |
| Q_1_ | | 394.04 - 549.32 | 14/ 68 | | 1*** | 1*** |
| Q_2_ | | 549.32 - 606.47 | 18/ 65 | | 1.35 (0.62-2.93); 0.454 | 1.39 (0.62-3.13); 0.430 |
| Q_3_ | | 606.47 - 661.80 | 17/ 65 | | 1.27 (0.58-2.79); 0.550 | 1.31 (0.58-2.95); 0.518 |
| Q_4_ | | 661.80 - 3238.90 | 17/ 66 | | 1.25 (0.57-2.74); 0.576 | 1.36 (0.61-3.05); 0.452 |
| Iron (Fe) | |  |  | |  |  |
| Q_1_ | | 217.55 - 765.54 | 16/ 66 | | 1.10 (0.53-2.40); 0.813 | 1.13 (0.51-2.52); 0.757 |
| Q_2_ | | 765.54 - 960.61 | 17/ 65 | | 1.19 (0.55-2.57); 0.666 | 1.24 (0.56-2.74); 0.593 |
| Q_3_ | | 960.61 - 1202.71 | 17/ 65 | | 1.19 (0.55-2.57); 0.666 | 1.30 (0.59-2.87); 0.519 |
| Q_4_ | | 1202.71 - 2102.20 | 15/ 68 | | 1*** | 1*** |
| **Normal BMI**  **(N = 145) #** | | **Se levels (µg/L)!** |  | |  |  |
| Q_1_ | | 42.68 - 58.27 | 11/ 25 | | 3.63 (1.03-12.76); **0.044** | 4.79 (1.13-20.26); **0.033** |
| Q_2_ | | 58.27 - 61.86 | 8/ 28 | | 2.36 (0.64-8.66); 0.197 | 3.00 (0.74-12.28); 0.126 |
| Q_3_ | | 61.86 - 67.69 | 4/ 32 | | 1.03 (0.24-4.48); 0.967 | 1.46 (0.30-7.09); 0.635 |
| Q_4_ | | 67.69 - 89.17 | 4/ 33 | | 1*** | 1*** |
| **BMI ≥ 25**  **(N = 180) #** | | **Se levels (µg/L)!** |  | |  |  |
| Q_1_ | | 44.39 - 54.24 | 9/ 36 | | 1.36 (0.46-4.03); 0,.582 | 1.46 (0.48-4.49); 0.506 |
| Q_2_ | | 54.24 - 58.91 | 11/ 34 | | 1.76 (0.61-5.04); 0.295 | 1.72 (0.58-5.11); 0.327 |
| Q_3_ | | 58.91 – 64.92 | 10/ 35 | | 1.55 (0.53-4.52); 0.421 | 1.58 (0.53-4.77); 0.415 |
| Q_4_ | | 64.92 – 125.54 | 7/ 38 | | 1*** | 1*** |
| **Male fetus**  **(N = 176)** | | **Se levels (µg/L)!** |  | |  |  |
| Q_1_ | | 44.39 – 56.32 | 16/ 28 | | 4.42 (1.36-14.34); **0.013** | 6.26 (1.79-22.05); **0.004** |
| Q_2_ | | 56.32 - 61.46 | 8/ 36 | | 1.82 (0.52-6.32); 0.347 | 1.97 (0.56-7.02); 0.293 |
| Q_3_ | | 61.46 - 67.32 | 8/ 36 | | 1.94 (0.55-6.83); 0.301 | 2.32 (0.64-8.45); 0.200 |
| Q_4_ | | 67.32 – 125.54 | 5/ 39 | | 1*** | 1*** |
| **Female fetus**  **(N = 154)** | | **Se levels (µg/L)!** |  | |  |  |
| Q_1_ | | 42.68 – 55.80 | 5/ 33 | | 1.03 (0.27-3.89); 0.965 | 1.20 (0.31-4.66); 0.791 |
| Q_2_ | | 55.80 - 60.41 | 10/ 29 | | 2.35 (0.72-7.65); 0.158 | 2.10 (0.62-7.18); 0.235 |
| Q_3_ | | 60.41 – 65.11 | 9/ 29 | | 2.11 (0.64-7.01); 0.223 | 2.20 (0.65-7.45); 0.205 |
| Q_4_ | | 65.11 – 109.57 | 5/ 34 | | 1*** | 1*** |

! Microelement concentrations were measured in the serum from the 10-14^th^ week and border values were included in lower quartile; *OR: crude odds ratios calculated in univariate logistic regression in the matched groups and AOR: adjusted odds ratio calculated in the multivariate logistic regression after adjusting for maternal height, gestational weight gain per week and prior fetal macrosomia; ** p- value obtained using the Wald test (p < 0.05 was considered to be significant); *** the reference quartile with the lowest number of LGA cases (OR = 1.00);

CI: confidence intervals; LGA cases (mothers delivering to newborn >90^th^ percentile); AGA controls (mothers delivering to newborn between 10 - 90^th^ percentile); BMI (kg/m²): pre-pregnancy body mass index.
