# Supplementary material for: Serum Microelements in Early Pregnancy and their Risk of Large-for-Gestational Age Birth Weight
Source: Nutrients. 2020 Mar 24;12(3):866. doi: 10.3390/nu12030866 (PMC7146262; doi:10.3390/nu12030866)
Supplement: Supplementary file 1 [file nutrients-12-00866-s001.zip › Table S4.docx]

**Table S4.** Characteristics of participants of two studies (that were based for data from the same cohort): for LGA and SGA risk.

|  | **Study for LGA risk** | **Study for SGA risk** |  |
| --- | --- | --- | --- |
|  | Mean (SD) or n (%) | Mean (SD) or n (%) |  |
| **Characteristics** | **Whole group**  **(N = 330)** | **Whole group**  **(N = 240)** | **p ***** |
| Maternal age (years) | 35.8 (3.7) | 35.4 (4.7) | SI |
| Gestational age at recruitment | 12.1 (0.9) | 12.1 (0.9) | SI |
| Pre-pregnancy BMI kg/m² | 26.4 (4.9) | 25.2 (4.6) | **0.004** |
| Gestational age at delivery | 38.7 (1.9) | 38.4 (2.1) | SI |
| Birth weight | 3483.0 (578.3) | 3113.6 (619.6) | **<0.001** |
| Primiparous | 107 (32.4 %) | 97 (40.4 %) | SI |
| Pre-pregnancy BMI ≥25 kg/m² | 180 (54.6 %) | 100 (41.7 %) | **0.002** |
| Smokers * | 14 (4.2 %) | 22 (9.2 %) | **0.017** |
| FA in I trimester | 106 (32.1 %) | 77 (32.1 %) | SI |
| Multivitamins in II-III trimester | 167 (50.6 %) | 112 (46.7 %) | SI |
| PIH cases | 64 (19.4 %) | 53 (22.1 %) | SI |
| GDM cases | 76 (23.0 %) | 49 (20.4 %) | SI |
| Fetal sex / son | 176 (53.3 %) | 125 (52.1 %) | SI |
| **Selenium concentrations (µg/L) **** |  |  |  |
| Whole group | 61.72 (8.92) | 61.95 (7.81) | SI |
| Cases | 59.79 (6.48) | 59.60 (8.60) | SI |
| AGA controls | 62.20 (9.37) | 62.54 (7.50) | SI |
|  | **0.036 ** **** | **0.020 ** **** |  |
| Pre-pregnancy BMI ≥25 kg/m² | 60.41 (9.39) | 60.20 (7.43) | SI |
| Pre-pregnancy BMI 18.5-24.99 kg/m² | 63.10 (7.10) | 63.05 (7.92) | SI |
|  | **<0.001 ** **** | **0.005 ** **** |  |
| Smokers * | 58.27 (5.97) | 57.13 (7.16) | SI |
| Women who had never smoked | 61.96 (9.32) | 62.45 (7.83) | SI |
|  | SI ** ** | **0.005** **** **** |  |
| **Characteristics** | **LGA cases**  **(n = 66)** | **SGA cases**  **(N = 48)** | **P ***** |
| Maternal age (years) | 36.0 (3.6) | 35.5 (4.7) | SI |
| Gestational age at recruitment | 12.0 (0.8) | 12.0 (0.9) | SI |
| Pre-pregnancy BMI kg/m² | 27.1 (5.4) | 24.9 (4.8) | **<0.001** |
| Gestational age at delivery | 38.8 (1.1) | 37.9 (2.6) | SI |
| Birth weight | 4174.1 (254.1) | 2358.0 (511.1) | **<0.001** |
| Primiparous | 20 (30.3) | 19 (39.6 %) | SI |
| Pre-pregnancy BMI ≥25 kg/m² | 37 (56.1 %) | 18 (37.5 %) | **<0.001** |
| Smokers * | - | 10 (20.8 %) | - |
| FA in I trimester | 19 (28.8 %) | 13 (27.1 %) | SI |
| Multivitamins in II-III trimester | 33 (50.0 %) | 23 (47.9 %) | SI |
| Male fetus | 37(56.1 %) | 25 (52.1 %) | SI |
| PIH cases | 18 (27.3 %) | 22 (45.8 %) | **0.040** |
| GDM cases | 20 (30.3 %) | 10 (20.8 %) | **0.007** |
| **Selenium concentrations (µg/L) **** |  |  |  |
| All cases | 59.79 (6.48) | 59.60 (8.60) | SI |
| Pre-pregnancy BMI ≥25 kg/m² | 59.94 (6.21) | 59.22 (7.17) | SI |
| Pre-pregnancy BMI: 18.5-24.99 kg/m² | 60.01 (6.55) | 59.05 (9.11) | SI |
|  | SI ** ** | SI ** ** |  |
| Smokers * | - | 56.27 (7.73) | - |
| Women who had never smoked | 60.20 (6.53) | 60.63 (8.95) | SI |
|  | -** ** | SI ** ** |  |

* Smokers at the beginning of pregnancy; ** Selenium concentrations were measured in serum from the 10-14-th gestational week; *** The Mann-Whitney U test was used for comparisons of continuous variables and the Pearson chi-square test was used for categorical variables comparisons (p-value < 0.05 was considered to be significant); ** **comparisons of Se concentrations between the subgroups; SI: statistically insignificant
