# Supplementary material for: Serum Microelements in Early Pregnancy and their Risk of Large-for-Gestational Age Birth Weight
Source: Nutrients. 2020 Mar 24;12(3):866. doi: 10.3390/nu12030866 (PMC7146262; doi:10.3390/nu12030866)
Supplement: Supplementary file 1 [file nutrients-12-00866-s001.zip › Figure S1.docx]

**
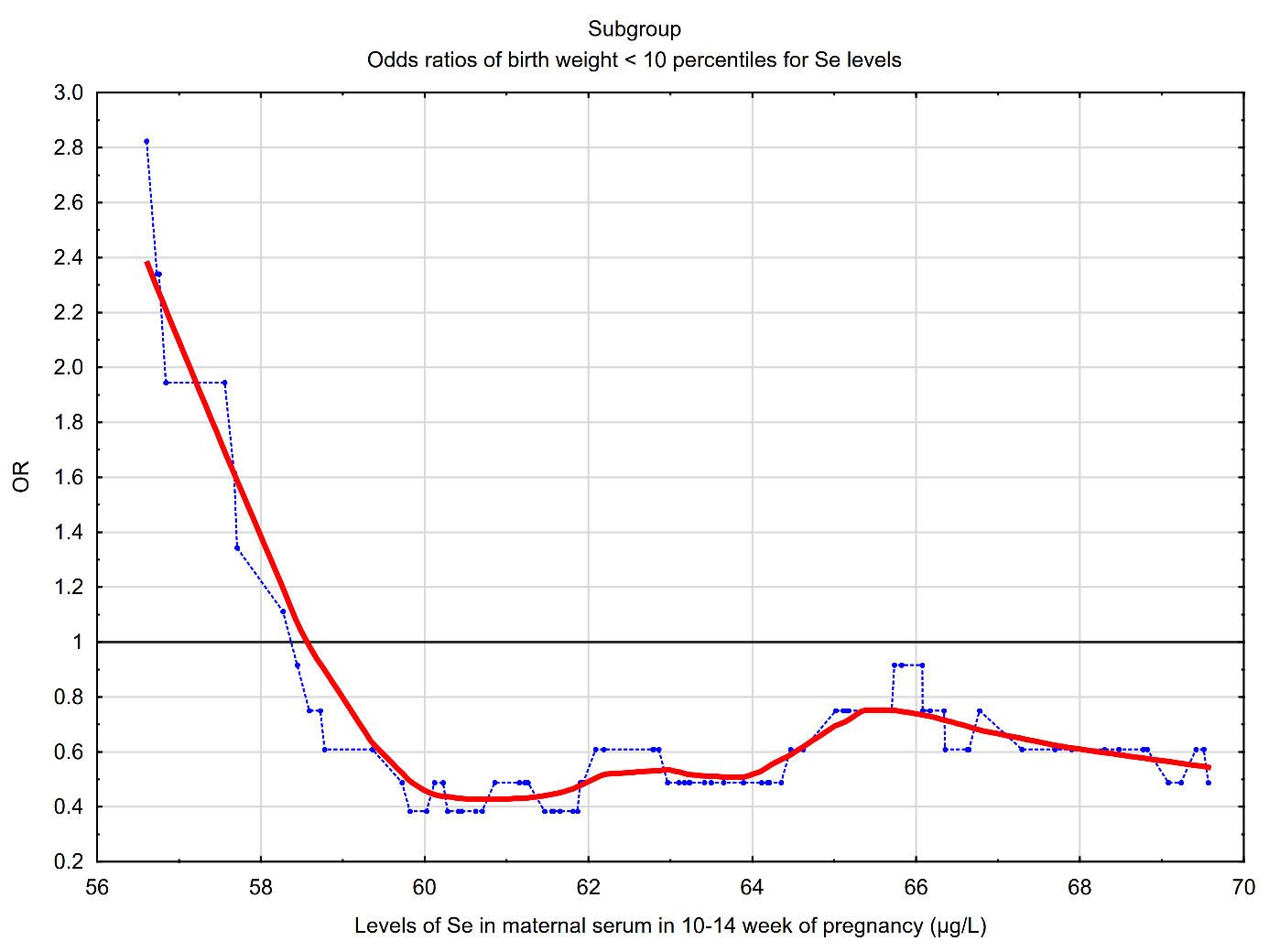
**

**Figure S1.**The risk profile of SGA birth weight (small-for-gestational age birth weight; <10th percentile) for early pregnancy maternal serum selenium (Se) concentrations in the subgroup of women with the normal pre-pregnancy BMI (N = 134) (the results were obtained in our previous case-control study based on the results of the same cohort). The graph shows the changes in the odds ratio of SGA calculated on a sliding window with respect to the changes in the Se concentrations. The window width adopted was 30 observations. The blue points correspond to the odds ratios (OR) of SGA in a window containing a fixed number of neighboring cases (the center of the window is for a given value of Se concentration). The red curve represents the LGA risk profile smoothed with the Lowess method. The horizontal black line is the reference line for OR = 1; the points above the line indicate an increased risk.
