# Supplementary material for: Serum Microelements in Early Pregnancy and their Risk of Large-for-Gestational Age Birth Weight
Source: Nutrients. 2020 Mar 24;12(3):866. doi: 10.3390/nu12030866 (PMC7146262; doi:10.3390/nu12030866)
Supplement: Supplementary file 1 [file nutrients-12-00866-s001.zip › Table S1.docx]

**Table S1.** The characteristics of microelements’ concentrations in the AGA and LGA group, in the subgroup of pre-pregnancy BMI ≥25 kg/m² and the women with a female fetus.

|  | **Controls**  **(AGA group)*** | **Cases**  **(LGA group)*** |  |
| --- | --- | --- | --- |
| **Microelements (µg/L) **** | **Mean (SD)** | **Mean (SD)** | **p***** |
| Pre-pregnancy BMI ≥25 kg/m²  (N = 180) | n = 143 | n = 37 |  |
| Selenium (Se) | 60.53 (6.65) | 59.94 (6.21) | 0.992 |
| Zinc (Zn) | 637.59 (252.57) | 631.82 (83.96) | 0.171 |
| Copper (Cu) | 1883.84 (371.21) | 1808.51 (319.51) | 0.376 |
| Iron (Fe) | 977.88 (353.10) | 939.05 (276.98) | 0.814 |
| Women with a female fetus  (N = 154) | n = 125 | n = 29 |  |
| Selenium (Se) | 61.17 (9.31) | 60.13 (6.00) | 0.778 |
| Zinc (Zn) | 634.53 (260.62) | 604.48 (77.35) | 0.828 |
| Copper (Cu) | 1809.88 (321.22) | 1697.87 (328.11) | 0.095 |
| Iron (Fe) | 976.58 (340.29) | 984.26 (241.50) | 0.641 |

*AGA: appropriate-for-gestational age birth weight (10-90^th^ percentile), LGA: large-for-gestational age birth weight (>90^th^ percentile); ** Microelements were measured in maternal serum from the 10-14-th gestational week; *** The Mann-Whitney U test was used and medians were compared (*p*-value < 0.05 was considered to be significant); Normal pre-pregnancy BMI: body mass index 18.5 – 24.99 kg/m².
